# PROBING THE COMPLEX INTERACTIONS BETWEEN DIET, DISEASE, AND AGING

**DOI:** 10.1093/geroni/igz038.261

**Published:** 2019-11-08

**Authors:** Rozalyn Anderson

**Affiliations:** University of Wisconsin Madison, Madison, United States

## Abstract

Nutrient response pathways are conserved modifiers of longevity, and dietary restriction is the most studied intervention for slowing aging in laboratory animals. For many years it was believed that lifespan extension from dietary restriction was tightly linked to total caloric intake. Recent evidence suggests that the interaction between diet and aging is more complex than this, however, with nutrient sensing, dietary composition, and circadian components all playing a role. This symposium will delve into some of the complex biological interactions linking food intake, lifespan, and diseases of aging.

